# Supplementary material for: Prior antibiotics and risk of subsequent Herpes zoster: A population-based case control study
Source: PLoS One. 2022 Oct 27;17(10):e0276807. doi: 10.1371/journal.pone.0276807 (PMC9612511; doi:10.1371/journal.pone.0276807)
Supplement: S1 Appendix — (DOCX) [file pone.0276807.s001.docx]

| **Appendix 1 – Herpes zoster codes**  Medical code Read term  390 Herpes zoster  516 Shingles  7331 Ramsey Hunt Syndrome  8936 Ophthalmic herpes zoster infection  14718 Herpes zoster with ophthalmic complication  14793 Herpes zoster otitis externa  18918 Herpes zoster ophthalmicus  21069 Herpes zoster with unspecified complication  21471 Herpes zoster NOS  25320 Herpes zoster with dermatitis of eyelid  27403 Geniculate herpes zoster  27546 Herpes zoster with keratoconjunctivitis  31681 Herpes zoster - otitis externa  33810 Herpes zoster with other ophthalmic complication  38531 Herpes zoster with other specified complication NOS  39692 Polyneuropathy in herpes zoster  43235 Herpes zoster with other specified complication  44944 Herpes zoster with meningitis  47375 Zoster encephalitis  50537 Herpes zoster with other CNS complications  51692 Encephalitis due to herpes zoster  52126 Herpes zoster with other central nervous system complication  52319 Disseminated zoster  55940 Herpes zoster iridocyclitis  57895 Herpes zoster meningitis  62558 Infective otitis externa due to herpes zoster  63739 Herpes zoster with other CNS complication NOS  69405 Herpes zoster encephalitis  70197 [X]Zoster without complications  71464 Meningitis due to herpes zoster virus  **Appendix 2 – Zoster vaccination codes**   \| Medical code \| Read code \| Read term \| \| --- \| --- \| --- \| \| 106904 \| 65FY.00 \| Herpes zoster vaccination \| \| 106593 \| 65FY.11 \| Shingles vaccination \| \| 107067 \| 65FY000 \| Herpes zoster vaccination given by other health care provide \| \| 106948 \| 68Nv.00 \| No consent for herpes zoster vaccination \| \| 106946 \| 8I2r.00 \| Herpes zoster vaccination contraindicated \| \| 106947 \| 8IEl.00 \| Herpes zoster vaccination declined \| \| 107061 \| 9Nig.00 \| Did not attend herpes zoster vaccination \| \| 108895 \| U60K600 \| [X]Herpes zoster vacc caus adverse effects therapeutic use \|  \| Product code \| \| \| Product name \| \| --- \| --- \| --- \| --- \| \| 47327 \| \| \| Zostavax vaccine powder and solvent for suspension for injection 0.65ml pre-filled syringes (sanofi pasteur MSD Ltd) \| \| 48314 \| \| \| Shingles (Herpes Zoster) vaccine (live) powder and solvent for suspension for injection 0.65ml pre-filled syringes \| \|  \| \| \|  \| \| Immunology \| \| Description \| \| \| 88 \| Shingles \| \| \| \| 91 \| Shingles OHP \| \| \| |
| --- | --- | --- | --- | --- | --- | --- | --- | --- | --- | --- | --- | --- | --- | --- | --- | --- | --- | --- | --- | --- | --- | --- | --- | --- | --- | --- | --- | --- | --- | --- | --- | --- | --- | --- | --- | --- | --- | --- | --- | --- | --- | --- | --- | --- | --- | --- | --- | --- | --- | --- | --- | --- | --- | --- | --- |
