# Supplementary material for: Prior antibiotics and risk of subsequent Herpes zoster: A population-based case control study
Source: PLoS One. 2022 Oct 27;17(10):e0276807. doi: 10.1371/journal.pone.0276807 (PMC9612511; doi:10.1371/journal.pone.0276807)
Supplement: Attachment — Submitted filename: POSone response to reviewers v5.docx [file pone.0276807.s002.docx]

Response to reviewers’ comments

PONE-D-21-26046

**Antibiotic driven dysregulated immune response: a case control study of Herpes zoster**

(The effect of antibiotics on the immune response: a case control study of Herpes zoster incidence)

Thank you for the reviewers’ helpful comments. We have tried to address them all as described below. Please note the change in title, prompted by a reviewer’s comment.

**I read that the matching variables are age, sex, family practice and index date for HZV. Why did authors not adjust for cohort date entry? Are they sure that cases and controls have the same length of follow-up?**

**In case control studies it is essential that cases and matched controls have the same length of period in which exposure is assessed however this is not clear from the paper. The authors must specify this issue, if cases and matched control have different length of period in which exposure is assessed the results may be biased.**

As noted in the Method section, the reference time period for our study was from time of HZV diagnosis (controls were allocated the index date for cases) backward to age 18 (e.g., lifetime time period) in both cases and controls. A key assumption of case-control studies is for controls to be selected independent of exposure, and thus we respectfully disagree with the Reviewer suggestion for similar length of exposure. Following recent suggestions (Keogh et al., 2016: https://bmcmedresmethodol.biomedcentral.com/articles/10.1186/s12874-015-0104-0) we have estimated time-varying exposure-association regression (fitted regression analyses within successive time periods) to address potential variation in exposure over time between cases and controls. Within case-control studies it is not possible to estimate incidence of the outcome (which is dependent on time), rather the probability (odds) of disease in cases (exposed) vs controls (non-exposed), over the entire study period (age 5 to Zoster in our study).). Further, the control group were matched on year of birth, as well as the GP practice, ensuring that the distribution of the exposure status was the same in the absence of an exposure disease association (null hypothesis). Please also note, that we have used incidence density sampling (to minimise differential loss to follow-up), whereby a case could still be a control for an earlier case – we have clarified in the text that we have used incidence density sampling for controls selection.

**The authors must explain why they chose as exposure the time since first antibiotic use and not the time since last antibiotic use**

We in fact examined both and found little difference, as shown in the Figure below. While both choices can denote sensitive periods, first prescription is likely to represent chronic rather than acute exposure to antibiotics. Also, we excluded prescription in the previous 12 months to address possible protopathic bias though, as mentioned in the text, sensitivity analyses that included the previous 12 months showed similar results.

**Provide an explanation of the results obtained from a biological point of view.**

This was an epidemiological study that did not examine biological mechanisms. Nevertheless, we hypothesised that the viral reactivation was the result of antibiotics damaging the microbiome and interfering with the immune response. We have added more discussion on the plausibility of this hypothesis.

**How is possible that use of antibiotics can be associated with Herpes zoster onset after 10 year? Maybe it would be better to study an acute effect of antibiotics?**

As noted above, we do not know the underlying mechanism but there is evidence that antibiotics can cause long term microbiome damage. No doubt antibiotics also have acute effects, but these were more difficult to study in our study design given the possibility of protopathic bias, that is early manifestations of HZV resulted in an antibiotic being prescribed. That is why we excluded antibiotics prescribed a year prior to the HZV diagnosis. We hypothesised that the impact of antibiotics on HZV is gradual through microbiome dysbiosis accompanied or not with low grade persistent inflammation which require repeated exposure to adverse events over longer periods of time to reduce the immune response ability to fight infections.

**In the title there is the word ‘incidence’. How is it possible to calculate ‘incidence’ with a case control study?**

We apologise for this error – we have changed the title to: "Antibiotic driven dysregulated immune response: a case control study of Herpes zoster”

**I saw, in each figure, the “overall effect”. How did the authors calculate it? I think with a meta-analysis model (indeed in figure 5 the I2 is showed). Is it possible to include this method in the “statistical analysis” section?**

This has been clarified in the Methods/Statistical Analysis section. We now state that ‘*We have used the metan command in Stata to estimate the overall effect size and related heterogeneity statistic (I^2^) for HVZ across the different classifications of the study exposure (antibiotics) within each estimation model’.*

**I would move the sentence “..Since the findings….these results here.” from “statistical analysis” to “Results”.**

We have moved this sentence to the Results section.

**There is only a parenthesis in line 97 section “Statistical analysis”.**

This stray bracket has now been removed.

**In the introduction, when you type "A recent meta analysis…..4 milion cases of HZV" you must insert the corresponding reference and write: "A recent meta analysis…..4 milion cases of HZV (ref Marra et al)…."**

We have moved the relevant reference as suggested. We have kept the format in Vancouver style, as per journal standards.

**In Fig. 1 you write that the study involves 175335 patients with HZ and 313647 patients without EZ. However, in Tab 1 I read 163754 subjects with HZ and 331559 controls. Can you explain this discrepancy?**

We apologise for the errors. We have now rectified the Flow chart shown in Figure 1 to reflect the correct figures included in the analysis as in Table 1.

**In Tab. 1 there is an empty line corresponding to the asthma variable. Are they missing values or zero values?**

We have now excluded the line as asthma was not associated with the study outcomes, and we have decided to include the most pragmatic estimation model.

**In Tab 1 I read for example LES 509 (0) where zero would represent the ratio 509/163754. I would recommend replacing the value 0 with a more correct <1 or << 1**

We have edited the table accordingly.

**In Fig. 5 you report the value of I^2^ without any comment in the text. If you report the value of I^2^ with the associated probability, you should clearly specify what these values entail**

We have now included the following text in the results section: ‘According to the **I^2^** statistics, there was significant heterogeneity (p<0.001) between the type of antibiotics and the HZV.’

**The last sentence “The effect of antibiotics on the immune..” appears to be little justified in the context and therefore superfluous.**

We have changed this sentence to reflect the possible effect of antibiotics on other viral diseases in general.

In the covering letter ….

***Please upload your study’s minimal underlying data set as either Supporting Information files or to a stable, public repository and include the relevant URLs, DOIs, or accession numbers within your revised cover letter.***

We have now clarified data availability (that was not included when the files were transferred from PLOS Medicine). The study is based on data extracted from the UK’s Clinical Practice Research Datalink. Under the terms of our access, we are unable to make our data set publicly available but the data can be extracted (ie our study replicated) from CPRD after approval by their ethics committee and payment of the requisite fee. We have also included the following statement in the document: ‘CPRD data accessed under the protocol number 20_134. Codes used to identify cases and for data analysis are available from the authors’.
