# Supplementary material for: Prior antibiotics and risk of subsequent Herpes zoster: A population-based case control study
Source: PLoS One. 2022 Oct 27;17(10):e0276807. doi: 10.1371/journal.pone.0276807 (PMC9612511; doi:10.1371/journal.pone.0276807)
Supplement: Attachment — Submitted filename: Response to reviewers R.docx [file pone.0276807.s003.docx]

PONE-D-21-26046R1

**Response to reviewers**

**Thank you for your detailed and very helpful comments. We have attended to them all as described below.**

Please review your reference list to ensure that it is complete and correct. If you have cited papers that have been retracted, please include the rationale for doing so in the manuscript text, or remove these references and replace them with relevant current references. Any changes to the reference list should be mentioned in the rebuttal letter that accompanies your revised manuscript. If you need to cite a retracted article, indicate the article’s retracted status in the References list and also include a citation and full reference for the retraction notice.

**The reference list has been updated as it is a considerable time since the paper was written.**

“Antibiotic driven dysregulated immune response” and the short title “The effect of antibiotics on the immune response” should be revised as they do not reflect the actual content of the current analysis. Immune response has not been evaluated in the current study. Please revise for consistency with the research study you have performed.

**We have revised the title to:** **The association of prior antibiotics with Herpes zoster: a case control study**

- Throughout the text, when mentioning CIs, please refer to them as 95%CIs.

- Please revise “previous varicella infection” to “previous varicella zoster virus infection”

- Please mention the measurement unit i.e., years, here: “18 to 50”

- Is it quite uncommon to use the name “Herpes zoster virus infection” and the abbreviation HZV. More commonly, the term “varicella zoster virus” is used, abbreviated as VZV, while the disease per se is abbreviated as HZ.

- Please revise “varicella reactivation” to “varicella zoster virus reactivation”.

**All of these changes have been made as requested.**

- “this study has shown that antibiotics may be involved in the reactivation of the varicella virus through their effect on the immune system”. This is not a direct conclusion of the study as you have not directly studied causality and neither immune responses. Please revise for consistency with the actual data that you presented.

**Revised**: *By finding an association between prior antibiotics and Herpes zoster this study has shown that antibiotics may be involved in the reactivation of the varicella zoster virus.*

- “A test of this hypothesis occurs when a patient is given broad spectrum antibiotics for some unrelated condition” – please revise this statement in the text. This does not provide confirmatory proof that this hypothesis is absolutely true.

**Revised**: *The possible association of Herpes zoster, which involves a reactivation of a previous varicella zoster virus infection, with prior antibiotic exposure might indicate a potential link with the immune response.*

- “established medical diagnostic codes” is quite vague. Please clarify at least which coding system was used, if not the actual codes used for the database query.

**The coding system was Read/SNOMED CT and this is now described in the Method section. We have also included a list of the codes used as an Appendix.**

- “the number of antimicrobial prescriptions issued during the follow-up period (i.e., 0, 1, 2, 3, 4, 5, 6-10, >10 years)” If this represents “the number”, then why is the measurement unit in years? Please clarify in the text that you are referring to the number of antibiotic prescriptions broken down by follow-up periods.

**Clarified that we are referring to the number of antibiotic prescriptions broken down by follow-up periods.**

- Please revise “HIV” to “HIV infection”.

**Revised.**

- Why was the 12 months range chosen here instead of a shorter range? “Prescriptions issued 12 months before the diagnosis were excluded from the analysis to reduce the risk of protopathic bias.”

**An exclusion period of either 6 months or 1 year is often chosen. We consider that a shorter timeframe has a higher chance of the risk of protopathic bias (an antibiotic being prescribed for an initial sign/symptom of a yet undiagnosed HZ infection), that may overemphasize the risk in the exposed (antibiotics) group, and consequently overestimate the risk of HZ associated with prior antibiotics prescribing. To be on the conservative side we therefore selected a 1 year period. This is now described in the Method section.**

- Please check decimal points for CIs here: “aOR of 1.30 (CIs 122-137) to penicillins with an aOR of 1.87 (CIs 184-190)”.

**Corrected.**

- From Fig 1 it isn’t entirely clear when matching was performed, before or after the first exclusionary step when 27848 cases of HZ were excluded because of missing data.

**Figure 1 has now been amended to clarify when matching occurred.**

- Also, in Fig 1, it is hard to follow and understand the numbers in the following box: “69420 excluded because missing data or cases with missing data/55659 no cases”. You are probably referring to controls excluded because you had to also exclude cases? Maybe a table legend could clarify this for the readers.

**We have chosen to use the Reviewer’s first suggestion for clarifying the statement in Figure 1. We have thus revised the relevant text in Figure 1 to clarify that: “..(55659 controls excluded due to the exclusion of corresponding cases)”.**

- Table 1: Please reconsider the title of the column “Herpes HZV”, potentially change it to “Herpes zoster”.

**Changed.**

- Table 1: Mean and SD are generally reported with the ± sign, to avoid confusion with median (IQR).

**Changed.**

- Fig 2: The figure title stated “previous 10+ years” while the graph lists 10 years. Please revise where appropriate, for consistency.

**Thank you. We have now revised the graph title to say the previous 10+ years, similar to the figure title and estimation model.**

- The results from Fig 3 should be discussed in more depth, since the ORs are gradually decreasing with more recent antibiotic treatment. This is somewhat counterintuitive, and the potential underlying mechanisms should be discussed into more depth. Furthermore, if not accurately clarified in the manuscript’s text, it could also be inadvertently misinterpreted as: prescribing antibiotics more recently could decrease the risk for HZ.

**We have addressed this in the Discussion (see below).**

- Fig 5: Please give more information on antibiotics for UTIs. Have you classified here antibiotics with local action only in the lower urinary tract, i.e., fosfomycin, nitrofurantoin, or also antibiotics with systemic action used for upper UTIs?

**We have classified antibiotics for both lower and upper UTIs together, in order to increase the study power but also to reflect the fact that antibiotics are likely to affect the microbiome regardless of indication.**

- The explanation that you have provided to the reviewer’s prior query: “The authors must explain why they chose as exposure the time since first antibiotic use and not the time since last antibiotic use” should be added to the manuscript.

**Please note that we did justify the selection of the former in the Discussion section (see below comment). Due to the implemented sensitivity analyses, using the time since last antibiotic would have affected the estimates from the protopathic bias-based analyses. However, we did examine both, time since first and last prescription but omitted the latter as it had limited impact on the direction of association. Further, we opted for the former in line with our hypothesis that longer exposure to antibiotics would be more likely to have a greater impact on microbiome dysbiosis.**

- More in depth discussion is needed for the following statement: “the finding that a first prescription more than 10 years prior to the HZV episode carried the highest risk suggests that any effect of antibiotics was long term rather than short term.” First, it would be important to justify from a pathophysiological stand point why one antibiotic course prescribed 10 years previously would still be expected to have an impact on the current microbiome. Second, when looking only at the first prescription, there is the bias of missing out on subsequent prescriptions which might be more recent and more relevant for a new onset dysmicrobism as a trigger for HZ.

- Furthermore, you state yourself that following antibiotic use “some recovery seems to occur within a year or so in adults”, which does not fit well with the observation of higher risk with older prescriptions, unless more clearly explained.

**Thank you: we can see this was confusing. We have now removed discussion of recovery from the Introduction and have discussed this more extensively in the second paragraph of the Discussion. In summary, there appears to be ‘recovery’ of the microbiome within 6 months or so but this is to a new stable state and not a return to the original.**

- “The results of this study add support to the view that microbiome dysbiosis (in this case caused by prior antibiotics) has an effect on the immune response”. Actually, it does not, because you have not studied the underlying immune mechanisms and you have not proven causality and the pathophysiological pathway. Please revise to a more conservative tone, keeping in line with your study’s exact findings.

- “this study has shown through an association between HZV and antibiotics that the latter may be involved in that reactivation through their effect on the immune system”. It does not. Please see my comment above and rephrase.

**Both statements have been revised to a more conservative tone and we have added:** *Further, our data did not allow us to directly test the proposed mechanisms (e.g., microbiome dysbiosis), and future studies are needed to confirm a direct link between antibiotic prescribing with microbiome dysbiosis in patients with HZ.*

- Please add a paragraph discussing your study’s limitations, i.e., not being able to assess causality, underlying mechanisms, degree to which results are generalizable and to whom, etc.

**We have discussed limitations across two paragraphs, one describing data limitations and generalisability, the other the problem of confounding. These include:**

*CPRD, however, does not record antibiotic prescriptions given in hospital nor whether patients actually consumed their prescribed antibiotics. A further limitation is that while the study sample is representative of the UK population, the findings may not necessarily apply to other countries with different patterns of antibiotic prescription and populations which may be more or less susceptible to antibiotic damage.*

*The main limitation of the study, as with any case-control design, is possible confounding which prevents assuming causality. Some potential confounders were addressed by adjusting for several co-variates that could have influenced both prescribing and caseness. For example, HZ is more common in diabetes and rheumatoid arthritis so these were added as covariates (4,44) as were corticosteroids given their known effect on immunosuppression. Even so, an alternative explanation for the association of antibiotics with HZ is the role of being ‘run down’ or having general malaise. There is some evidence that stress makes HZ more likely (45–47) and presenting general malaise to the doctor might be more likely to attract an antibiotic prescription. Yet while this remains an alternative explanation, the timelines revealed in this study would require general malaise or stressors over ten years ago being managed with an antibiotic and for that malaise to continue to influence the appearance of HZ a decade later without significant additional antibiotics in that period. Residual confounding, however, still remains a possibility.*

Further responses

Reviewer #1: Althought the authors replied to the reviewers comments but I still have strong doubts.

1) In the "point by point" the authors write “…the reference time period for our study was from time of HZV diagnosis (controls were allocated the index date for cases) backward to age 18 (e.g., lifetime time period) in both cases and controls ..” this explanation does not fit with what is written in the manuscript.

**This statement was made in the context of describing, and defending, our case control design and we agree it does seem confusing. We believe that the description in the Method section is clearer.**

2) The authors stated in the “point by point” to use the extension of case-control of Keogh et al but the "method section" of the paper does not reflect the statistical analysis of the cited paper.

**The Keogh et al paper compared two analytic methods for case-control studies showing that both produced similar results. Please note that our statement reflected the implementation of the Keogh et al (2016) analysis to demonstrate the robustness of our study findings in response to the Reviewer’s concerns but have not included these results in the original paper as it produced similar findings.**

3) In the "point by point" the authors stated the type of matching is “frequency” however in the paper in the line XX the authors wrote that the matching is “individual”. Moreover, in the new version of the manuscript they report “Incidence density sampling was used to select controls to minimise differential loss to follow-up” however if this is a case-control study, there is no follow-up period but a look-back period.

**We cannot find any reference to ‘frequency’ in our earlier response so we are not sure how to respond. Please note that in the manuscript under the Study population we did state that: “Two controls were sought for each case, individually matched on age (within 5 years to ensure sufficient matched controls per practice), …“. We agree that ‘follow-up’ is not entirely appropriate for a (retrospective) case control study but it implies the period between patient ‘entry’ into the study and the index date, thus a retrospective follow-up.**

4) The model used for the meta-analysis is not clear. Did the authors use a fixed or random model?

**We have clarified that random-effects meta-analysis model was used to account for heterogeneity.**
